# Rib Motions Don’t Completely Hinge on Joint Design: Costal Joint Anatomy and Ventilatory Kinematics in a Teiid Lizard, *Salvator merianae*

**DOI:** 10.1093/iob/oby004

**Published:** 2019-01-02

**Authors:** J G Capano, S Moritz, R L Cieri, L Reveret, E L Brainerd

**Affiliations:** 1 Department of Ecology and Evolutionary Biology, Brown University, Providence, RI 02906, USA; 2 Department of Biology, Community College of Rhode Island, Warwick, RI 02886, USA; 3 School of Biological Sciences, University of Utah, Salt Lake City, UT 84112, USA; 4 Inria Grenoble Rhone Alpes, 655 Avenue de l’Europe, 38330 Montbonnot-Saint-Martin, France

## Abstract

Rib rotations contribute to lung ventilation in most extant amniotes. These rotations are typically described as bucket-handle rotation about a dorsoventral axis, caliper rotation about a craniocaudal axis, and pump-handle rotation about a mediolateral axis. A synapomorphy for Lepidosauria is single-headed costovertebral articulations derived from the ancestral double-headed articulations of most amniotes. With a single articular surface, the costovertebral joints of squamates have the potential to rotate with three degrees-of-freedom (DOFs), but considerable variation exists in joint shape. We compared the costovertebral morphology of the Argentine black and white tegu, *Salvator merianae*, with the green iguana, *Iguana iguana*, and found that the costovertebral articulations of *I. iguana* were hemispherical, while those of *S. merianae* were dorsoventrally elongated and hemiellipsoidal. We predicted that the elongate joints in *S. merianae* would permit bucket-handle rotations while restricting caliper and pump-handle rotations, relative to the rounded joints of *I. iguana*. We used X-ray reconstruction of moving morphology to quantify rib rotations during breathing in *S. merianae* for comparison with prior work in *I. iguana*. Consistent with our hypothesis, we found less caliper motion in *S. meriana*e than in *I. iguana*, but unexpectedly found similar pump-handle magnitudes in each species. The dorsoventrally elongate costovertebral morphology of *S. merianae* may provide passive rib support to reduce the conflict between locomotion and ventilation. Moreover, the observation of multiple DOFs during rib rotations in both species suggests that permissive costovertebral morphology may be more related to the biological roles of ribs outside of ventilation and help explain the evolution of this trait.

## Introduction

Costal aspiration, in which motions of the ribs are used to ventilate the lungs, is the ancestral mode of ventilation for amniotes (Janis and Keller 2001; Brainerd and Owerkowicz 2006). Rib motions continue to contribute to ventilation mechanisms in all extant amniotes except turtles, and although apparently simple, these movements are deceptively complex. Costal kinematics are three-dimensional (3D) rotations that result from a combination of joint articulations, rib shapes, and muscular arrangements, and variation within and among these factors can substantially influence the overall motions (De Troyer et al. 2005; Claessens 2009a, 2009b; Brainerd 2015; Brainerd et al. 2016; Brocklehurst et al. 2017). All amniotes appear to have evolved from an ancestor with double-headed ribs, i.e., bicapitate, composed of a tuberculum and a capitulum that articulate with a diapophysis and parapophysis on each vertebra (Romer 1956; Hoffstetter and Gasc 1969). Therefore, primitive amniotes appear to have relied primarily on rib motions about bicapitate costovertebral joints to ventilate their lungs (Janis and Keller 2001).

Squamate reptiles, i.e., lizards and snakes, retain this reliance on costal aspiration to ventilate their lungs, yet their costovertebral joint morphology is notably different from other amniotes. Squamates are unusual in having single-headed ribs, i.e., unicapitate, that articulate with a single articular surface on the vertebrae, the synapophysis (Romer 1956; Hoffstetter and Gasc 1969). Developmental evidence indicates that synapophyses are formed through fusion of the parapophysis and diapophysis of each vertebrae, rather than the loss of one of these structures (Romer 1956; Hoffstetter and Gasc 1969). In bicapitate ribs, the two distinct articular surfaces make it possible to predict an anatomically constrained axis of rotation, which can be defined by the axis that runs through the tuberculum and capitulum of each rib head (De Troyer et al. 2005; Claessens 2009a). The predictive qualities of bicapitate morphology have recently been used to predict the general *in vivo* rib kinematics during lung ventilation in the American alligator, *Alligator mississippiensis.* Even with appreciable anatomical axes present, however, these predictions tended to misestimate the relative contribution from the three potential axes of rotation (Brocklehurst et al. 2017).

**Fig. 1:**
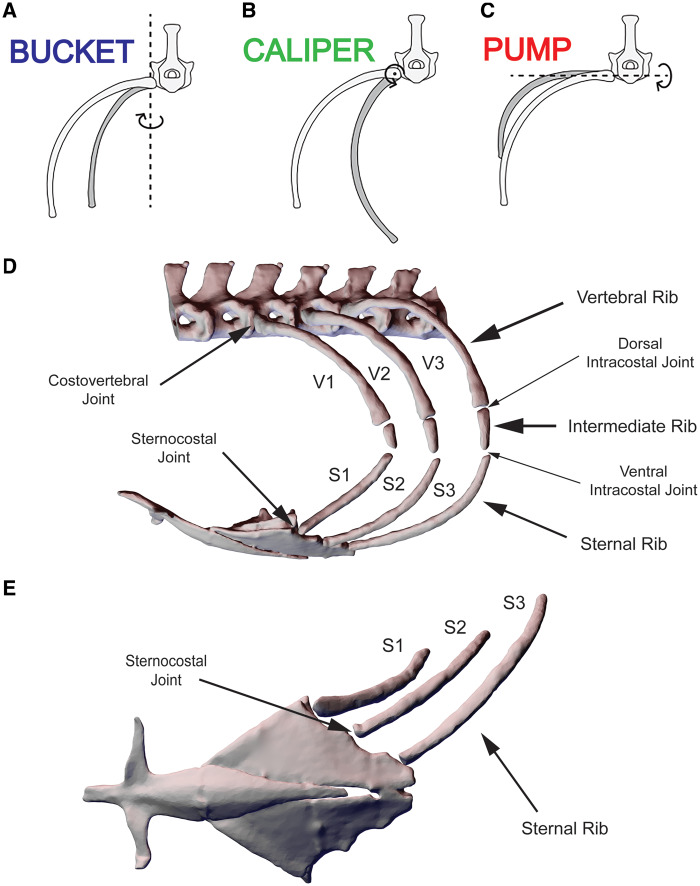
Costal kinematics and anatomy of the Argentine black and white tegu, *Salvator merianae.* Rib motions can be described as rotations about three anatomical axes: (**A**) bucket-handle rotation about a dorsoventral axis; (**B**) caliper rotation about a craniocaudal axis; and (**C**) pump-handle rotation about a mediolateral axis. (**D**) Anatomy of the thorax of *S. merianae*. (**E**) The sternal ribs articulate with the sternum via simple synchondrosis joints that suggest no anatomically constrained axis of rotation. Abbreviations: V1, V2, and V3, vertebral ribs 1, 2, and 3; S1, S2, and S3, sternal ribs 1, 2, and 3.

In the absence of double-headed articulations, the single-headed joints of squamates make it difficult to predict, *a priori*, about which axes the ribs will rotate during lung ventilation. Unicapitate costovertebral joints have no anticipated anatomical axis of rotation and therefore may permit motions in all three potential rotational degrees of freedom (DOF). These potential rib motions have typically been described as bucket-handle rotation, caliper rotation, and pump-handle rotation (Fig. 1A–C). While orientation of these rotations has not always been consistent, recent convention is to align the axes relative to the body axis (Brainerd et al. 2016; Brocklehurst et al. 2017). For this study, and to enable comparison between species, bucket-handle rotation is defined about a dorsoventral axis (Fig. 1A), caliper rotation is about a craniocaudal axis (Fig. 1B), and pump-handle rotation is about a mediolateral axis (Fig. 1C). With such potentially permissive rotations and morphology, the unicapitate ribs of squamates vary substantially from other amniotes and yet the functional role of this trait in the evolutionary history of Squamata has remained difficult to discern.

**Fig. 2:**
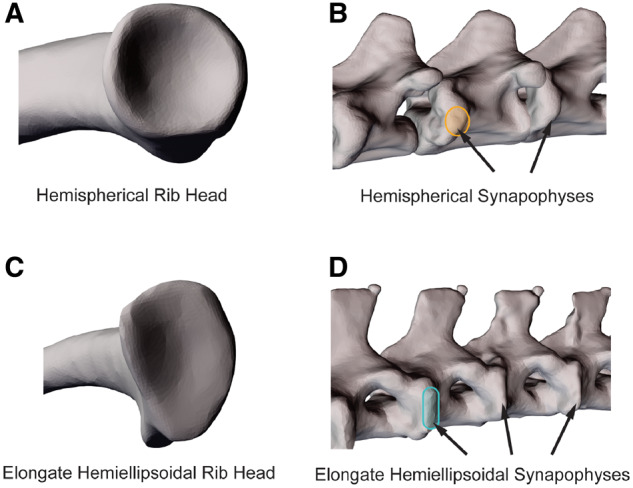
Comparison of costovertebral joint structure between *I. iguana* and *S. merianae*. The shape of the unicapitate costovertebral joints varies considerably between these species. (**A**) The rib heads of V1 in *I. iguana* are cup-shaped and articulate with (**B**) hemispherical, convex synapophyses (outlined in orange). In contrast, (**C**) the rib heads of V1 in *S. merianae* are elongated dorsoventrally and articulate with (**D**) similarly dorsoventrally elongate, hemiellipsoidal synapophyses (outlined in teal).

Costovertebral articulations in green iguanas, *Iguana iguana*, follow the typically described squamate pattern. The articular surface of each rib head is cup-shaped (Fig. 2A) and articulates with a hemispherical synapophysis on each vertebra (Fig. 2B), similar to a ball-and-socket joint (Hoffstetter and Gasc 1969; Brainerd 2015; Brainerd et al. 2016). The kinematics of these ribs during lung ventilation have been found to be primarily dominated by bucket-handle rotations, but considerable amounts of pump-handle and caliper rotation also occur (Brainerd 2015; Brainerd et al. 2016). Ecologically, green iguanas are a primarily arboreal and herbivorous species with a predominantly sedentary lifestyle; these tropical lizards rely on crypsis and immobility to avoid predation and infrequently stray far from their small core territories (Greene et al. 1978; Rand et al. 1989).

In contrast, costovertebral articulations in the Argentine black and white tegu, *Salvator merianae*, a teiid lizard species often sympatric with *I. iguana*, are markedly different. The articular surface of each rib head of *S. merianae* is dorsoventrally elongated (Fig. 2C) and articulates with a hemiellipsoidal synapophysis on each vertebra (Fig. 2D), reminiscent of a hinge joint. In contrast to green iguanas, Argentine black and white tegus are an omnivorous, cursorial lizard species with an active lifestyle; these strongly built opportunists forage widely in aquatic, terrestrial, and fossorial habitats throughout their extensive home ranges (Kiefer and Sazima 2002; Winck et al. 2011; Sazima and D’Angelo 2013).

The primary aim of this study is to determine whether varying unicapitate costovertebral morphologies constrain or permit the use of different axes of rotation during costal ventilation in these species. We use marker-based X-ray reconstruction of moving morphology (XROMM) (Brainerd et al. 2010; Knörlein et al. 2016) to analyze the skeletal kinematics of *S. merianae* during lung ventilation. We then compare the rib kinematics about the hinge-like joints of *S. merianae* to the previously studied rib kinematics about the ball-and-socket-like joints of *I. iguana* (Brainerd et al. 2016). Although the rib rotations of *I. iguana* were found to be dominated by bucket-handle rotations, substantial variation among individuals was also noted in the amount of caliper and pump-handle rotation observed (Brainerd et al. 2016). The dorsoventrally elongated costovertebral joints of *S. merianae* (Fig. 2C, D) appear to be more restrictive than the more rounded joints of *I. iguana* (Fig. 2A, B), with a potential dorsoventral axis of rotation anatomically determined by the hinge-like shape. We therefore predict that the rib rotations of *S. merianae* will be constrained about both craniocaudal and mediolateral axes, thereby minimizing caliper and pump-handle rotations and functioning like a hinge joint. These findings will further test the *a priori* capacity of joint morphology to predict *in vivo* kinematics and continue to inform the form–function relationship between costal joint morphology and rib kinematics. This work may also provide some insight into the perplexing evolution of unicapitate morphology at the base of Lepidosauria and the potential costal aspiration mechanisms of stem amniotes and other fossil taxa.

## Materials and methods

Three adult Argentine black and white tegus, *S.merianae* (Duméril and Bibron, 1839), were used throughout this study. The animals were moderately sized adults with body masses ranging from 1.2 to 2.1 kg, including two males (tegu03 and tegu06) and one female (tegu05). Animal care and experimental procedures were approved by the Institutional Animal Care and Use Committee of Brown University.

We used marker-based XROMM to create 3D animations of the vertebral column, ribs and sternum during lung ventilation. We followed the procedures for marker-based XROMM as detailed in prior publications (Brainerd et al. 2010). Animals were anesthetized with isoflurane and a minimum of three radio-opaque metal markers were surgically implanted into each bone of interest. Three different marker types were used depending on the bone. Ribs were marked with beads-on-posts: 1 mm tantalum beads (Bal-tec, Los Angeles, CA, USA) with laser-drilled holes that were then mounted on short (2–3 mm long) segments of 000-sized insect pins (0.25 mm diameter). Dorsal ribs 1 and 2 were marked in all three individuals, with dorsal rib 3 additionally marked in tegu03 and the caudal-most cervical rib (C5) marked in tegu05. Dorsal ribs consisted of vertebral and sternal elements, both of which were marked for each dorsal rib, whereas cervical ribs were only vertebral elements. The sternum was marked with 0.8 mm solid tantalum beads placed directly into the bone. A hand drill was used to bore a small hole into the bone of interest, with drill bit diameters of either 0.25 or 0.8 mm, dependent on marker type. The beads-on-posts and solid beads were then press-fitted into the drilled holes. Vertebrae were marked with a combination of tungsten carbide conical markers (Kambic et al. 2014) and press-fitted 0.8 mm tantalum beads. Markers were spaced as far apart as possible on each bone to maximize the accuracy of the rigid body bone animations (Brainerd et al. 2010).

All individuals recovered from surgical procedures for a minimum of 7 days before data acquisition began. Analgesics were administered immediately before surgery, immediately after surgery, and for a minimum of 3 days following surgical procedures. All animals resumed normal behavior and feeding regimes within 3 days and no further pain medication was required.

X-ray videos were recorded with custom biplanar videoradiography equipment (Miranda et al. 2011). Videos were recorded at 60 frames per second, with 70–80 kV and 250–400 mA. The video data used for this publication have been deposited in the XMAPortal (xmaportal.org) in the study “Tegu Lung Ventilation” with permanent ID BROWN29. All video data are stored in accordance with best practices for video data management in organismal biology (Brainerd et al. 2017).

After video data collection, animals were anesthetized, euthanized with sodium pentobarbital, and secondarily exsanguinated. Computed tomography (CT) scans of the trunks of all individuals were collected with a Fidex Animage veterinary scanner at Brown University (Fidex, Animage, Pleasanton, CA, USA, 110 kV, variable mA, 0.173 mm slice thickness). Open source medical imaging software, Horos (Purview, Annapolis, MD, USA), was used to produce 3D polygonal mesh surface models of each marked bone and the associated radio-opaque markers. This resulted in 3D mesh surface models of each bone and the bone’s position relative to the markers.

Additional CT scans were collected for morphological comparison of costovertebral joint structures. In these instances, each bone of interest was dissected from the specimen, visually assessed, and independently air-contrast scanned. This method ensured more clearly defined skeletal edges and provided visual dissection confirmation of morphological variation.

### Marker tracking and XROMM animation

Biplanar X-ray videos were analyzed with XMALab to track the locations of each marker. Marker tracking precision, measured as the mean standard deviation of pairwise marker-to-marker distances within rigid bodies (Brainerd et al. 2010) was 0.046 mm, with the single best pairwise s.d. being 0.015 mm and the worst being 0.129 mm. Rigid–body transformations were calculated in XMALab (Knörlein et al. 2016).

Surgical implantation of beads-on-posts proved technically difficult at times and some ribs were inadequately marked for marker-based XROMM tracking. In these instances, ribs either had too few markers, i.e., two rather than three markers due to misplacement or failure, or the markers were so co-linear that XMALab software was unable to accurately determine 3D rigid body orientation. In these cases, “virtual” markers were generated on the vertebrae, on the sternum, or at the ventral intracostal joint. Based on motion of fully marked vertebral and sternal ribs, it was apparent that little to no translation occurred at the costovertebral, sternocostal, or ventral intracostal joints. Therefore, the center of rotation about these joints is a fixed position between the rotating rigid bodies. We created a virtual marker at this center of rotation and then animated that virtual marker with a completely marked rigid body, e.g., the associated fully marked vertebra of a vertebral rib. This virtual marker served as an additional rigid body point to stabilize and orient the insufficiently marked bones. We then checked the accuracy of the virtual marker driven bones by importing X-ray videos into Maya and visually ensuring that the animated bone motions matched the X-ray video movements.

The tracked 3D coordinates for each marker were used to calculate rigid–body transformations that were then filtered with a Butterworth low-pass filter within XMALab (cut-off frequency, 1–2 Hz) (Knörlein et al. 2016). These rigid–body transformations were then applied in Maya animation software (Autodesk, San Rafael, CA, USA) to their associated 3D bone models. Six breathing cycles from full inhalation to full exhalation were animated for all three individuals. The first dorsal rib was animated for all individuals and various combinations of cervical and other dorsal ribs were additionally animated among individuals.

### Joint coordinate systems

**Fig. 3:**
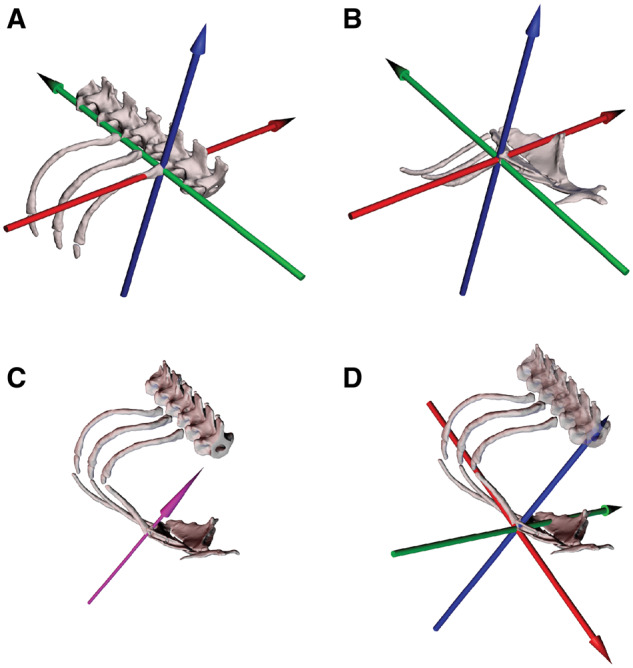
Costovertebral, sternocostal, and helically inspired sternocostal joint coordinate systems (JCSs). Dorsal is up and anterior is to lower right in all images. Anatomical coordinate systems are oriented such that *Z*-axis rotation (blue) is bucket-handle about a dorsoventral axis, *Y*-axis rotation (green) is caliper about a craniocaudal axis, and *X*-axis rotation (red) is pump-handle about a mediolateral axis. (**A**) Costovertebral JCS in oblique view; rib shown in reference pose. This JCS measures vertebral rib rotations and translations relative to the vertebral column. (**B**) Sternocostal JCS in oblique view; ribs shown in reference pose. This JCS measures sternal rib rotations and translations relative to the sternum. (**C**) HA orientation for the sternocostal joint from one breathing trial. The purple arrow represents the dominant HA for the sternal rib rigid body relative to the sternum rigid body as computed by the HA tool. The HA is calculated from the sternal rib motion in each trial and varies in orientation from trial to trial. (**D**) Helically inspired sternocostal JCS in oblique view. A JCS was oriented at the center of rotation of the sternocostal joint, with the *Z*-axis (blue) aligned to the angle of the HA and the *X*-axis (red) aligned along the long-axis of the sternal rib.

Skeletal kinematics were measured by applying joint coordinate systems (JCSs) to the XROMM animations produced in Maya (Maya XROMM Tools; xromm.org). These JCSs were applied to two different joints per rib in order to measure the translations and rotations of the vertebral rib relative to the vertebral column (Fig. 3A), and the sternal rib relative to the sternum (Fig. 3B). Rotations about each joint measured with JCSs describe these motions as Euler angles, with the rotation order set as ZYX. Each JCS was oriented so the *Z*-axis would describe the largest range of motion, followed by the *Y*-axis, and *X*-axis, following the best practice for rotation orders set forth by previous studies (Brainerd et al. 2010). After preliminary analysis of both vertebral and sternal ribs, the largest range of motion was in the bucket-handle rotation, so each *Z*-axis was oriented dorsoventrally to capture these rotations (Fig. 3). This rotation order is also the same as used in prior studies investigating rib kinematics during lung ventilation (Brainerd et al. 2016; Brocklehurst et al. 2017; Cieri et al. 2018) and allows for direct comparisons with other species.

Prior to animation, all bones were arranged in reference poses, similar to the reference poses developed for *I. iguana* (Brainerd et al. 2016). These poses were not naturally occurring positions, but were replicable orientations that allowed for consistent and repeatable comparison of rib positions and kinematics among different individuals (Baier et al. 2013; Brainerd et al. 2016). The vertebral ribs were oriented such that the neck of each rib lies tangential to a horizontal plane placed at the height of the costovertebral joint. The vertebral ribs were then positioned so that the neck was perpendicular to a vertical plane running through the center of the corresponding costovertebral joint and finally rotated so that the tip of the rib was just caudal to this plane (Fig. 3A). This replicable reference pose served as a zero position, with the vertebral rib perpendicular to the long-axis of the body and in an easily reproducible position relative to the vertebral column. Polarity of the rotations followed the right-hand rule, with exhalation being associated with decreasing or negative bucket-handle angles and inhalation associated with increasing or positive bucket-handle angles, i.e., rotation about a dorsoventral axis or *Z*-axis. Sternal ribs were similarly arranged into a reference “zero” position. The long-axis of the rib was oriented to be perpendicular to the long-axis of the body, where most of the rib lies in the transverse plane (Fig. 3B). Each rib was then rotated so that the distal tip of the rib was in the same horizontal plane at the height of the sternocostal joint.

The duration, magnitude, and mean rib posture of each breath varied within and among individuals. Breaths were compared by determining the maximum and minimum magnitudes of bucket-handle rotation for each rib in each trial. These peak values of bucket-handle rotation were used as maximum inhalation and maximum exhalation for each representative breath and the relative contributions of bucket-handle, caliper, and pump-handle rotations were assessed during this time interval. The absolute values of the magnitudes of each axis of rotation were summed at these peaks to account for total rotational magnitude in each event, i.e., caliper and pump-handle rotations were not consistently positive or negative whereas bucket-handle rotation was. Each axis’ magnitude was then divided by this summation to calculate the percent contribution from each axis to the specific breath’s rotations. Identical procedures were followed for the sternal ribs.

### Helically inspired sternocostal JCSs

We expect the sternal ribs of *S. merianae* to move with the vertebral ribs, given their anatomical linkage (Fig. 1D), but the sternal ribs may also show greater variations in their posture or motions due to their simple cartilaginous articulations with the sternum (Fig. 1E). Therefore, to capture this potential variation in the axis of rotation of the sternal ribs, we used JCSs inspired by helical axis (HA) orientations in addition to the anatomically oriented sternocostal JCS described above (Fig. 3B). These helically inspired JCSs allowed us to visualize and quantify the dominant axis of rotation for each breath and compare the orientations of each HA to assess the variation in sternocostal rotations. The HA, also called a screw axis, is a mathematical construction that represents motion as rotation and translation about a single axis that varies in orientation and position over time (Woltring et al. 1985). For each time step in a kinematic event, the calculated unit vector orientation (HA orientation) simultaneously represents the single axis about which a rigid body rotates and the line along which it translates. We developed an interactive HA tool, implemented in Autodesk Maya, to analyze each breath. This tool provides an estimate of the 3D axis of the instant angular velocity using finite time difference: relative motion about the sternocostal joint is represented as the 3D displacement between forward and backward rigid body positions in animation time with respect to a center frame. This defines the center and width of the time window used for finite time difference. Each trial was initially analyzed with the HA tool to quantify the frame at which the sternal rib reached the highest instantaneous velocity for the breathing event, i.e., the frame where the HA most closely approximates the center of rotation for the joint. This frame was then used as our center frame input. The HA tool allows the width of the time window to be set as a user-adjustable parameter. For this parameter, there is a trade-off between being too large a value that could then miss a single rotation axis motion, and too small a value that does not show enough rotational motion, thereby producing instability in the computation of the HA. Breathing motions were relatively slow events (inhalation took approximately 800 ms per breath) and thus required considerably more time than the typical frame window used for other HA analyses. The time window used in this study was set to 25% of the frame window of each inhalation, as measured from maximum exhalation to maximum inhalation. This percent of the frame window provided enough motion for robust HA calculation while being easily replicable among trials and individuals. These procedures were followed for the first sternal rib of all breathing trials analyzed for each of the three individuals of *S. merianae*.

The results of the HA calculations produced a dominant axis that best described rotation of the sternal rib about the sternocostal joint relative to the sternum for each individual trial (and varied in orientation from trial to trial). We used the orientation of this axis to inspire the placement of JCSs at the center frame calculated with the HA tool. In Maya, the JCS was point-snapped to the center of rotation of the HA and the *Z*-axis was point-oriented to align with the unit vector orientation of the HA (Fig. 3C). Helically inspired JCSs were then manually rotated about the *Z*-axis to align the *X*-axis of the JCS with the long-axis of the sternal rib at the center frame. The polarity of the rotations of all helically inspired JCSs followed the right-hand rule as outlined for anatomical JCSs (Fig. 3D).

### Data and statistical analysis

Prior to in-depth analysis, we needed to determine whether there was significant kinematic variation between each marked rib along the thorax of *S. merianae*. Raw rotational magnitude variations between the marked ribs were analyzed in two ways. Ribs that were marked in all individuals were checked with a two-way ANOVA to account for both individual and rib variation. Ribs that were marked in a single individual were checked with a one-way ANOVA.

Subsequent rib rotation analyses compared the relative magnitude changes between maximum exhalation and maximum inhalation for each of the three axes of rotation for the first dorsal rib. Raw rotational magnitudes were used to quantify the relative contribution from each axis of rotation, i.e., the percent contribution of each axis’ magnitude to total rib rotational magnitude from maximum exhalation to maximum inhalation. For species comparisons, relative contributions of the two species were analyzed with a nested ANOVA to determine differences associated with species while taking individual variation into account. All statistical analyses were performed in JMP Pro (version 13.2; SAS Institute). Differences were considered statistically significant at the level of *P* ≤ 0.05.

## Results

### Costal morphology

The presacral axial skeleton of *S. merianae* consists of 8 cervical vertebrae and 16 dorsal vertebrae. The first three cervical vertebrae do not carry ribs while cervical vertebrae 4, 5, and 6 have short floating ribs. Cervical vertebrae 7 and 8 have long, well-developed ribs that end in a cartilaginous plug.

The first three dorsal ribs of *S. merianae* that articulate with the sternum consist of three segments: an osseous vertebral rib, a cartilaginous intermediate rib, and a cartilaginous sternal rib (Fig. 1D). The osseous vertebral rib articulates with the cartilaginous intermediate rib at a short cartilaginous dorsal intracostal joint. The intermediate rib then articulates with the cartilaginous sternal rib at a ventral intracostal joint that appears in dissections to be a narrow cartilaginous “elbow” between the two segments. Manipulation of post-mortem specimens and excised ribs has shown that relative motion is potentially possible at both dorsal and ventral intracostal joints. Intermediate and sternal ribs are mineralized to the extent that they appear as independent segments in both videofluoroscopy and CT scans (Fig. 2D).

We also compared the costovertebral articulations of *S. merianae* to those of the previously studied green iguana, *I.iguana*, to investigate the influence of joint morphology on overall rib kinematics (see Carrier 1987, 1989, 1990; Brainerd et al. 2016, for anatomical descriptions of *I. iguana*). As noted in the “Introduction” section, the costovertebral articulations of *S. merianae* are dorsoventrally elongated and hemiellipsoidal and articulate with the vertebrae at elongate, hemiellipsoidal synapophyses (Fig. 2C, D). In contrast, the rib heads of *I. iguana* are cup-shaped and articulate with hemispherical convex surfaces on the vertebrae (Fig. 2A, B).

### Intracostal joint mobility

**Fig. 4:**
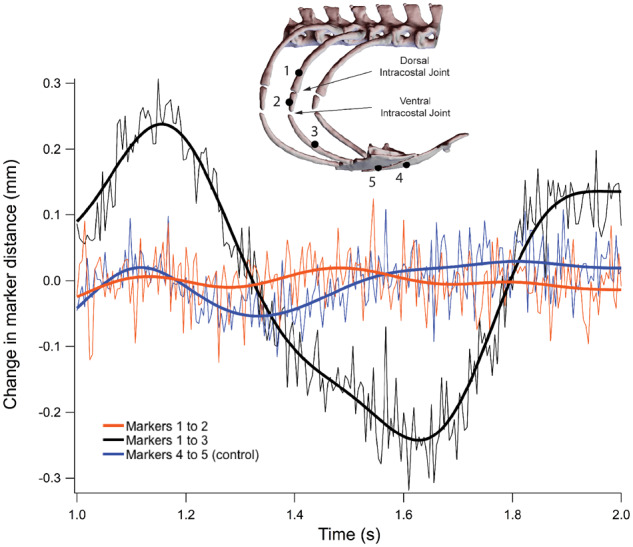
Intracostal joint mobility. Unfiltered and filtered marker-to-marker distance traces are shown. Markers 4 and 5 serve as a control because *a priori* no change in distance is expected within the sternum. A change in distance between markers 1 and 2 would indicate bending at the dorsal intracostal joint, but no bending was detected. The large change in distance between markers 1 and 3 indicates substantial bending at the ventral intracostal joint.

Rib segmentation in Argentine black and white tegus is tripartite (Fig. 1D), a condition found frequently within teiids and numerous other groups of squamates (Hoffstetter and Gasc 1969). Before we could start the XROMM animation process, we had to determine if and where measurable bending occurred between the three individual rib segments, the number of rigid bodies to animate, and whether individual segments flexed along their respective lengths. *A priori* we expected no significant deformation of the highly mineralized and rigid sternum during breathing, thus the pairwise distance changes between the markers in the sternum define our precision threshold as 0.05 mm (Fig. 4, markers 4 and 5). Pairwise distance changes between markers in the vertebral rib and intermediate rib showed no relative motion above the precision threshold (Fig. 4, markers 1 and 2). The slight fluctuations measured between markers 1 and 2 are within our marker tracking precision for this study (±0.1 mm) and indicate that no motion occurs about the dorsal intracostal joint, i.e., the vertebral and intermediate rib move as a single rigid body. Pairwise distance changes between markers in the vertebral rib and sternal rib (Fig. 4, markers 1 and 3) and the intermediate rib and sternal rib (data not shown, markers 2 and 3) showed significant relative motion. This indicates that the vertebral–intermediate unit moves relative to the sternal rib and that motion occurs about the ventral intracostal joint. Distance changes between markers within the sternal ribs were also within the ±0.1 mm precision and indicate that the sternal ribs also acted as rigid bodies (data not shown).

We then checked our rigid body assumptions by importing the original X-ray videos and MayaCam calibrations into Maya and visually ensuring that the animated bone motions matched the X-ray video movements. No evidence of flexion was observed for vertebral, intermediate, or sternal rib segments. Visual assessment also confirmed that no motion occurred between vertebral and intermediate ribs at the dorsal intracostal joint, while motion did occur between the intermediate and sternal ribs at the ventral intracostal joint. Based on all of these data, we henceforth treat the functional vertebral rib as the rigid vertebral–intermediate unit segment above the ventral intracostal joint, and the functional sternal rib as the rigid cartilaginous rib below the ventral intracostal joint.

### Costovertebral joint kinematics in *S. merianae* compared with *I. iguana*

In *S. merianae*, we found that rib rotations about the costovertebral joint were primarily dominated by bucket-handle rotations and that there was little variation in rib kinematics between the first vertebral rib (V1) and the other marked vertebral ribs, i.e., vertebral rib two (V2) in all three individuals, vertebral rib three (V3) in tegu03, and cervical rib five (C5) in tegu05. There was no significant difference in bucket-handle rotation between V1 and V2 in any individual (*P* = 0.9117), between V1, V2, and V3 in tegu03 (*P* = 0.9834), or between C5, V1, and V2 in tegu05 (*P* = 0.3493). We therefore decided to focus on V1, as the rotations were not substantially different for the other ribs and allowed for direct comparisons between individuals and species.

We found that rotation of the first vertebral rib (V1) about the costovertebral joint was predominantly bucket-handle rotation (blue, *Z*-axis) (mean ± s.e.m = 21.5 ± 1.8 deg) when measured with the anatomical JCS (Fig. 3A). The rib rotated caudally during exhalation and cranially during inhalation, effectively folding back and decreasing the volume of the thorax and then rotating forward and increasing the volume of the thorax. The costovertebral motions of the other two axes of rotation, caliper (green, *Y*-axis) (mean ± s.e.m = 2.1 ± 0.4 deg) and pump-handle (red, *X*-axis) (mean ± s.e.m = 4.7 ± 0.5 deg), were substantially smaller than the bucket-handle rotation in all individuals and in all trials.

The V1 rotations measured in our tegus were then analyzed by quantifying the relative contribution from each axis of rotation for all three individuals, with six breaths for each individual. The relative contribution from each axis of rotation was evaluated as the percent contribution to total rib rotation, i.e., overall magnitude changes from maximum exhalation to maximum inhalation (Fig. 6). The V1 rotations of *S. merianae* were dominated by bucket-handle rotation (mean ± s.e.m = 75 ± 1.8%), with substantially less contribution from caliper rotation (mean ± s.e.m = 7 ± 1.0%), and some contribution from pump-handle rotation (mean ± s.e.m = 18 ± 2.0%). The V1 rotations of previously published data on *I. iguana* (Brainerd et al. 2016) were also analyzed to quantify the relative contribution from each axis of rotation for four individuals, with five breaths per individual. We found that the V1 rotations of *I. iguana* were dominated by bucket-handle rotation (mean ± s.e.m = 69 ± 2.5%), with considerable contribution from caliper rotation (mean ± s.e.m = 19 ± 2.5%), and some contribution from pump-handle rotation (mean ± s.e.m = 12 ± 1.6%). These data were then compared with those collected for *S. merianae* in the current study and analyzed with a nested ANOVA. We found that *S. merianae* had significantly higher relative contribution from bucket-handle rotation than *I. iguana* for all breaths (*P* = 0.0301). *Salvatormerianae* also had significantly less contribution from caliper rotation (*P* < 0.0001) and significantly more contribution from pump-handle rotation (*P* < 0.0125) for all breaths when compared with those in *I. iguana*.

### Postural variation of the first vertebral rib

**Fig. 5:**
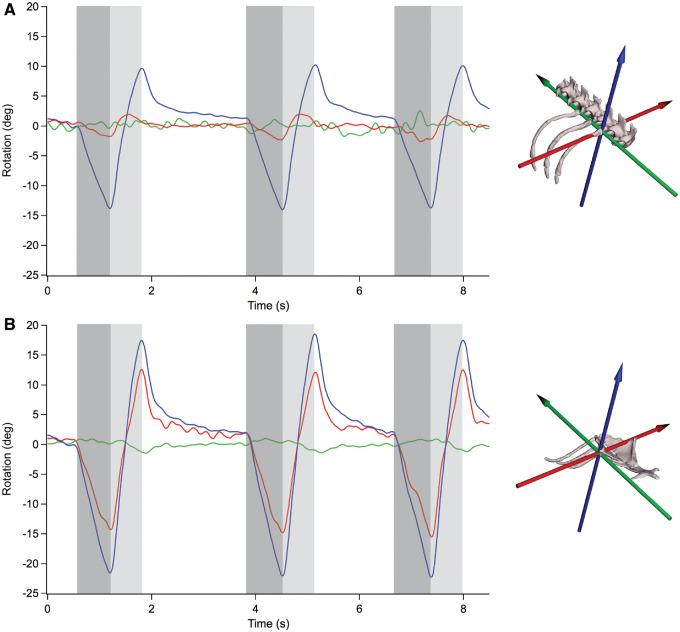
Euler angle rotations during deep breaths for costovertebral and sternocostal joints of dorsal rib 1. All angles are zeroed at their mean values. (**A**) Filtered Euler angle rotations from a representative breath for V1 at the costovertebral joint. Motion is dominated by bucket-handle rotation about a dorsoventral axis (blue); costovertebral JCS reference orientation shown. (**B**) Filtered Euler angle rotations from the same representative breath for S1 at the sternocostal joint. Substantial bucket-handle and pump-handle rotations contribute to sternal rib motion in many breaths; sternocostal JCS reference orientation shown. All data are from the same trial and representative breath from tegu03. Darker gray bars indicate the start and end of exhalation; lighter gray bars indicate the start and end of inhalation.

**Fig. 6:**
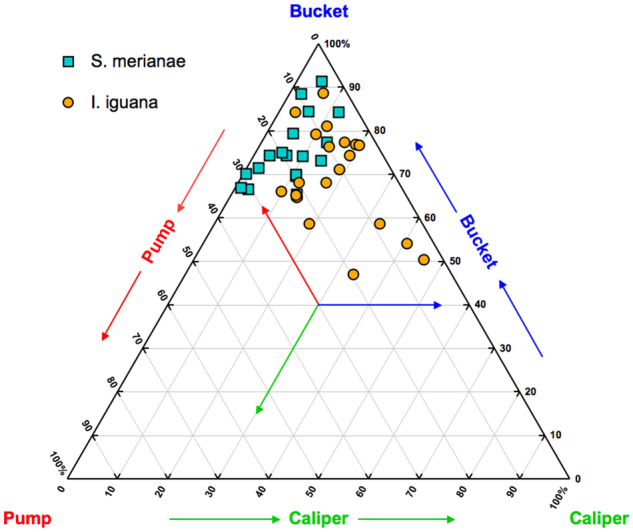
Species comparison of relative contribution from each axis of rotation during lung ventilation for V1. The rotations compared are from V1 from *S. merianae* (teal squares) and *I. iguana* (orange circles). The ternary diagram displays the relative percentage each axis contributes to the overall magnitude changes in vertebral rib rotation for representative breaths from each individual and trial (*S. merianae N* = 3 with six trials per individual; *I. iguana N* = 4 with five trials per individual). Each corner represents 100% contribution from the corresponding axis of rotation, and each point is one breath.

**Fig. 7:**
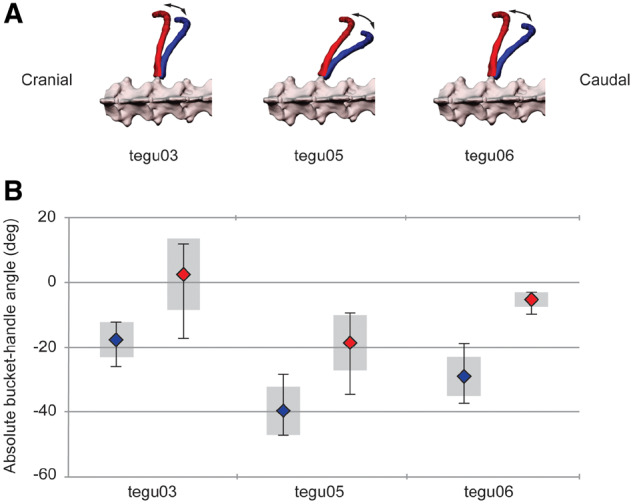
Variation in vertebral rib posture among individuals. The three tegus centered their rib rotations about different positions within their potential range of bucket-handle angles. (**A**) V1 rib positions at maximum inspiration (red) and maximum expiration (blue) for all three individuals. (**B**) Box plots of bucket-handle angles for V1 at maximum inspiration (red) and maximum expiration (blue) for each individual (*N* = 6 breaths per individual). Diamonds represent mean angles across all trials; whiskers indicate the range; shaded boxes indicate ±1 sd.

The results presented above are based on the magnitude of rotations for V1, i.e., the difference between maximum exhalation and maximum inhalation, with the angles either zeroed at their mean values (Fig. 5A) or represented as a fraction of 100% rotation or magnitude change (Fig. 6). This use of relative contribution allows for comparison between species but obscures the variation among individuals in actual rib posture and position. Visualization of the rib angles relative to the reference or “zero” posture reveals the differences in rib posture between individuals (Fig. 7). Bucket-handle rotation was the dominant axis of rotation and our three tegus used different positions within the potential range of bucket-handle angles: tegu05 and tegu06 breathed at more exhaled postures (deflated) while tegu03 breathed at more inhaled (inflated) postures. In fact, the mean exhaled posture of tegu03 (−18.8 deg) was closer to zero than the mean inhaled posture of tegu05 (−19.8 deg). Tegu03 even exceeded the “zero” posture and had inhaled rib rotations that were more cranial than the perpendicular “zero” reference position. Tegu03 and tegu05 also showed more variation in the range of rib postures at maximum inhalation (ranges of about 8–11 deg versus a range of about 2.5 deg in tegu06), while variation in rib posture range at maximum exhalation was similar among all individuals (ranges of about 5–7 deg). Compared with the breathing postures observed in *I. iguana*, *S. merianae* used significantly more inhaled rib postures for both maximum inhalation and maximum exhalation (*P* < 0.0001 in both instances).

### Sternocostal joint morphology and kinematics

**Fig. 8:**
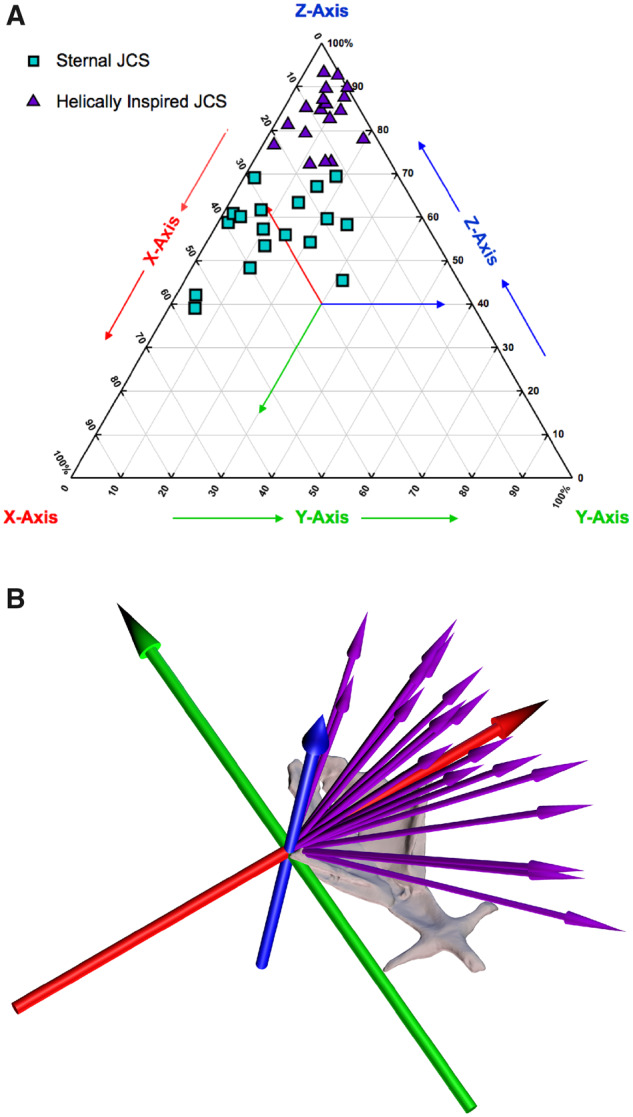
Sternal rib 1 rotations in *S. merianae* measured with an anatomical JCS versus helically inspired JCSs. (**A**) Comparison of the relative percentage each axis contributes to the overall magnitude changes for sternal rib 1 in *S. merianae* when measured with an anatomical JCS (teal squares) or helically inspired JCSs that vary from trial to trial (purple triangles). Note that motion is captured almost entirely in a single axis of rotation (*Z*-axis) when measured with helically inspired JCSs. (**B**) Visual comparison of the anatomically oriented JCS (large blue, green, and red axis system) with the helical axes computed for each individual breathing trial (purple arrows). The HA that best captures the sternocostal joint motion varies substantially from trial to trial.

The sternocostal joints of *S. merianae* are simple synchondroses whose anatomy suggests no anatomically oriented or constrained axis of rotation (Fig. 1E). We initially measured motion of the first sternal rib (S1) relative to the body axis using the conventional anatomical JCS (Fig. 3B). We found that the rotation of S1 about the sternocostal joint was composed of variable amounts of all three possible axes of rotation and that considerable variation was present within individual tegus and between trials (Fig. 8A; teal squares). We also found that motion was never dominated by a single axis of rotation but was often almost equally composed of bucket-handle and pump-handle rotations (Fig. 5B). We therefore anticipated that the dominant axis of rotation for the sternocostal joint would lie at some point between these two prevalent axes, but we were unable to readily quantify and compare the orientation of the anticipated intermediate axes using only anatomically oriented JCSs.

We therefore analyzed the motion of S1 about the sternocostal joint of *S. merianae* using helical axes and identified the dominant axis of rotation for all trials from all individuals (see the “Materials and methods” section; Fig. 8B). This analysis provided visual and quantitative comparisons of the orientation of the dominant axis for each breath. The unit vector orientations of the dominant HAs were found to cluster in a quadrant between the three anatomical axes, but no consistent pattern was found in the variation among these HAs, regardless of trial or individual (Fig. 8B). Sternal rib kinematics were then measured with a JCS inspired by the orientation of these dominant HAs (Fig. 3C, D). When measured with helically inspired JCSs, we determined that motion of S1 could be described almost entirely as rotation about a single axis, defined as the *Z*-axis (blue), for all trials from all individuals (Fig. 8A). When compared with the anatomical JCS measurements, we found that the helically inspired JCS measurements showed more relative contribution from the *Z*-axis (blue), less contribution from the *Y*-axis (green), and less contribution from the *X*-axis (red).

## Discussion

*A priori* we predicted that the dorsoventrally elongated and hemiellipsoidal costovertebral morphology of *S. merianae* would function as a hinge-like joint and constrain both caliper and pump-handle rotations (Figs. 1 and 2). As predicted, we observed that rotations about the costovertebral joints of *S. merianae* did not use substantial caliper rotations, but we also found more pump-handle rotation than anticipated. It is possible that these elongated unicapitate costovertebral joints may function to reduce the negative influences of locomotion on lung ventilation, functionally analogous to the constrained bicapitate costovertebral joints of other amniotes (Claessens 2015). Comparison of the kinematics of *S. merianae* with *I. iguana* revealed that the hemispherical articulations of *I. iguana* permitted significantly higher rotational freedom than *S. merianae*, specifically caliper rotations.

### Costovertebral morphology and vertebral rib kinematics

Although unicapitate costovertebral articulations are a synapomorphy for Lepidosauria, there appears to be considerable morphological variation in this trait. Unicapitate costovertebral joints are derived from the ancestral bicapitate condition for amniotes and an interesting intermediate condition between these two states still exists in the sister group to Squamata, the Rhynchocephalia. In *Sphenodon*, the only extant rhynchocephalian, the first few cervical ribs retain bicapitate morphology, while the remaining ribs are unicapitate, but with elongated oblong articulations (Hoffstetter and Gasc 1969). A similar morphology is found in other extant squamates (see numerous illustrations in Hoffstetter and Gasc 1969), specifically in *Varanus*, in which the anterior cervical ribs of some members also exhibit bicapitate morphology that then transitions to oblong costovertebral articulations in posterior regions (Hoffstetter and Gasc 1969). Interestingly, these descriptions for both *Sphenodon* and *Varanus* are similar to the hemiellipsoidal joints observed in *S. merianae* (Fig. 2C, D) and are in stark contrast to the hemispherical ball-and-socket-like articulations of *I. iguana* (Fig. 2A, B). Although unicapitate morphology is a synapomorphy for Lepidosauria, there appears to be a large degree of morphological variation within Squamata and the evolutionary transition and function of this variation remains perplexing. What then is the functional implication of this costovertebral joint diversity?

We have found that the hemiellipsoidal and hemispherical shapes of costovertebral articulations may influence the use of caliper rotation during lung ventilation. In this study, the more robust, hinge-like joints of *S. merianae*, when compared with those of *I. iguana*, exhibited significantly higher relative bucket-handle rotations and significantly lower relative caliper rotations (Fig. 6). Conforming to our *a priori* predictions, this suggests that the hemiellipsoidal joint shape of *S. merianae*, and therefore potentially *Sphenodon* and *Varanus*, may restrict extensive caliper rotations. When comparing the motions of *S. merianae* with those produced by the hemispherical joints of *I. iguana*, we determined that the ball-and-socket-like articulations appear to permit significantly higher caliper rotation (Fig. 6). It is important to note, however, that all of the rib kinematics observed were voluntary motions recorded during deep lung ventilation at rest and likely don’t represent the maximum range of motion permitted about each joint. Overall, our findings suggest that elongated unicapitate costovertebral joints may restrict caliper rotation, but further work is necessary to determine mechanistic causation.

Although *I. iguana* did show significantly higher caliper rotations than *S. merianae*, it is of interest that the motions of *I. iguana* were still predominantly composed of bucket-handle rotations. In *S. merianae* and many amniotes, the physical costovertebral joint might be anticipated to constrain rib rotations to a single axis of rotation (De Troyer et al. 2005; Claessens 2009b; Brocklehurst et al. 2017). The hemispherical, ball-and-socket-like costovertebral joints in *I. iguana* are less restrictive; therefore, the predominant use of bucket-handle rotations suggests that these motions are produced solely through musculature and connective tissue structures (Brainerd et al. 2016). If soft tissue can produce hinge-like motion, then what function do the restrictive joints of *S. merianae* serve? It is possible that the elongate articulations observed in *S. merianae* are not related solely to lung ventilation, but potentially to how the ribs participate in other behaviors in this species.

Ribs and their associated axial musculature serve important functions outside of breathing, particularly in locomotion. The sprawling posture of most lizards generates vertical and horizontal ground reaction forces during locomotion that are subsequently transmitted throughout the axial musculoskeletal system (Carrier 1987, 1990). The vertical component is stabilized by the epaxial musculature, whereas the horizontal component acts to produce long-axis torsion on the trunk (Carrier 1990; Ritter 1996). This torsional, rotational force is then transmitted to the ribs and the obliquely oriented intercostal muscles between them (Carrier 1987, 1990). As the lizard bends from side-to-side during locomotion, the obliquely oriented intercostal muscles unilaterally contract in rhythm with the motion to stabilize the trunk against these torsional forces (Carrier 1990). Therefore, as a lizard runs, the intercostals that are typically responsible for ventilation are instead co-opted to stabilize the trunk and thereby produce a conflict between locomotion and lung ventilation (Carrier 1989, 1990). This dual function consequently inhibits lung ventilation during vigorous locomotion and is a constraint still experienced throughout most of Squamata (Carrier 1987; Wang et al. 1997; Owerkowicz et al. 1999). Interestingly, *S. merianae* employ an unusually active foraging mode for squamates; these highly cursorial lizards actively search for food across diverse habitats throughout extensive home ranges (Kiefer and Sazima 2002; Sazima and D’Angelo 2013). In contrast, *I. iguana* are predominantly sedentary, foraging in trees and relying on crypsis and only brief bouts of activity to avoid predation (Greene et al. 1978). While the axial conflict between ventilation and locomotion is ancestral for Amniota, numerous muscular and skeletal innovations have evolved to reduce the negative effects of locomotion on ventilation, including modifications of costovertebral morphology (Brainerd and Owerkowicz 2006; Claessens 2015).

In numerous Archosaurs, restrictive costovertebral morphology has been suggested to help transmit and dissipate locomotor forces away from the rib cage in order to mitigate the influence of locomotion on ventilation (Claessens 2015). In the sprawling gait of crocodilians, locomotor impact forces are transmitted into relatively few vertebrae near the forelimbs due to the limited caudal extension of their pectoral girdle (Claessens 2015). Although costovertebral articulations of crocodilians are bicapitate, their orientation differs substantially along the trunk (Schachner et al. 2011). On vertebrae near the shoulders, the two rib heads are dorsoventrally oriented, whereas in posterior regions away from the forelimbs, the rib heads are mediolaterally oriented (Brocklehurst et al. 2017). This dorsoventral alignment in anterior regions has been hypothesized to function as a skeletal strut to resist the locomotor forces experienced in this pectoral region, whereas the mediolaterally oriented caudal regions are suggested to have evolved in response to ventilation mechanics (Schachner et al. 2009, 2011; Claessens 2015). Interestingly, these ribs nearest the pectoral girdle also participate little in ventilation and may therefore serve a primarily locomotor role (Brocklehurst et al. 2017). In birds, elongated scapulae transmit the locomotor forces associated with powered flight into a much greater number of thoracic vertebrae than crocodilians. Subsequently, thoracic vertebrae throughout the length of birds have their parapophyses directly ventral to their diapophyses. This too produces a strut that would help reduce the large locomotor forces transmitted onto the rib cage via the swinging motion of the forelimb during flight (Claessens 2015). Each of these costovertebral joints are suggested to help dissipate forces away from the rib cage and reduce the negative impact of locomotion on ventilation, thereby increasing locomotor capacity. Although interesting and compelling ideas, future work is necessary to validate these hypotheses and quantify the capacity of dorsoventrally oriented costovertebral joints to mitigate the reaction forces of locomotion.

**Fig. 9:**
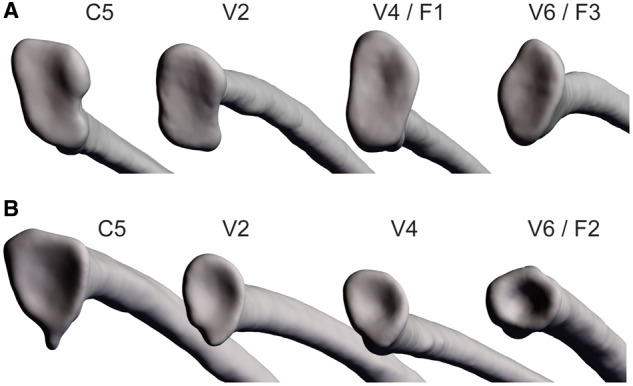
Comparison of craniocaudal variation in costovertebral joint shape between *S. merianae* and *I. iguana*. The shape of the unicapitate costovertebral joints of these species varies depending on their craniocaudal position along the trunk. (**A**) In *S. merianae*, the costovertebral joints closest to the pectoral girdle, particularly C5, V1, and V2, are more robust and become less elongate and robust caudally. (**B**) In *I. iguana*, the costovertebral joints are also somewhat more robust near the pectoral girdle, but not as robust as in *S. merianae*, and are relatively small and hemispherical throughout the rest of the trunk. Cranial is to the left, caudal is to the right. Abbreviations: C5, cervical rib five; V2–V6, vertebral ribs 2–6; F1-3, floating ribs 1-3.

It is therefore possible that the restrictive costovertebral morphology of *S. merianae* may be involved in locomotion and help support their cursorial lifestyle. The costovertebral joints of *S. merianae*, although unicapitate rather than bicapitate, are hemiellipsoidal and dorsoventrally elongated, similar to the dorsoventrally oriented bicapitate rib heads of both crocodilians and birds. The restrictive joints of *S. merianae* appear to restrict caliper rotation, at least during resting lung ventilation, the same DOF anticipated to be limited by a dorsoventrally oriented bicapitate strut. The hemiellipsoidal joints of *S. merianae* could potentially function to dissipate dorsoventrally oriented locomotor reaction forces, reduce the negative impact of locomotion on ventilation, and enable more cursoriality. Similar to the trend observed in crocodilians, we observed larger, more robust hemiellipsoidal articulations in the cervical and anterior vertebral ribs nearest the pectoral girdle of *S. merianae*, the region of the thorax where the reaction forces of locomotion would be highest (Fig. 9A). Comparatively, costovertebral articulations in the highly sedentary *I. iguana* are not as robust in anterior pectoral girdle regions and are relatively small throughout the trunk (Fig. 9B). Therefore, the costovertebral morphology of *S. merianae* may function to transmit locomotor forces away from the rib cage and enable ventilation at relatively higher locomotor speeds than in species without such robust joints, but measurements of rib kinematics during locomotion are necessary to validate this hypothesis (Boggs 2002).

While costovertebral morphology may function to dissipate locomotor forces in *S. merianae*, dorsoventrally elongate costovertebral articulations appear to occur throughout Lepidosauria. Joint morphologies similar to *S. merianae* have been observed in *Sphenodon*, *Varanus*, and various members of Serpentes (Hoffstetter and Gasc 1969). However, the wide disparity in the ecologies and locomotor strategies of these taxa make it difficult to interpret the connection between costovertebral morphology and locomotion; *Sphenodon* are relatively inactive, *Varanus* are active predators analogous to *S. merianae*, while snakes range from highly sedentary to active foragers (Hoffstetter and Gasc 1969; Greene 1997; Owerkowicz et al. 1999; Klein et al. 2000; Klein et al. 2003a). In the relatively inactive *Sphenodon*, do the dorsoventrally elongate costovertebral joints also function to dissipate torsional locomotor forces? Or does their presence merely represent the evolutionary transition from bicapitate to unicapitate? In members of *Varanus*, elongate costovertebral joints may provide an analogous function as suggested for *S. merianae*; varanids have similar aerobically active ecologies as *S. merianae* and recent analyses have revealed that they rotate their vertebral ribs during lung ventilation almost exclusively with bucket-handle and/or pump-handle rotations, i.e., little to no caliper (Cieri et al. 2018). Although it remains unclear how extensive and robust elongate costovertebral articulations are in varanids, they may similarly reduce the reaction forces of locomotion and contribute to the cursorial ecology of these lizards. It is also possible, however, that members of *Varanus* are in fact less reliant on rib motions for ventilation during locomotion than teiids, because of their derived accessory ventilation mechanism, gular pumping. If this is the case, then the rib kinematics and costovertebral morphology observed in varanids may reflect variation in anatomy, physiology, or other ecological demands (Owerkowicz et al. 1999; Cieri et al. 2018). In numerous species of Serpentes, their costovertebral joints are also dorsoventrally elongated, but they interestingly possess two distinct articular surfaces (Hoffstetter and Gasc 1969). These double-headed ribs have at times been described as bicapitate, despite snakes’ evolution from ancestors that would have presumably possessed the unicapitate plesiomorphy of Squamata (Hoffstetter and Gasc 1969; Pattishall and Cundall 2008; Young and Kardong 2010; Streicher and Wiens 2017). This condition in snakes potentially represents secondary evolution of multiple articular surfaces from previously unicapitate morphology.

Nevertheless, the functional implications of elongate costovertebral joints in snakes are unclear. In the absence of limbs, the mechanics and forces associated with locomotion differ considerably from their limbed lizard ancestors. There is no pectoral or pelvic girdle to transmit torsional forces to the trunk, yet because snakes locomote on their ventral scutes, the propulsive forces of locomotion must be transmitted down the body wall and into the environment, likely through the ribs (Cundall 1987). Moreover, it is unclear how the ribs of snakes and their potential motions participate in the various forms of serpentine locomotion. Similar to other amniotes, snakes also continue to use rib motions to ventilate their lungs, but it remains unclear how locomotion and ventilation interact in their attenuate body form (Rosenberg 1973). Additionally, snakes use their numerous ribs for a variety of other behaviors, including swimming, gliding, digging, basking, and defensive displays (Greene 1997; Young and Morain 2003; Pattishall and Cundall 2008; Young and Kardong 2010; Socha 2011). The presence of elongate costovertebral morphology in snakes may thus be related to the mechanics of limblessness, the application of or resistance to the forces associated with serpentine locomotion, or the multitude of other behaviors during which snakes use their ribs. It therefore appears that variation in costovertebral morphology is extensive within Lepidosauria and while there is likely a link between joint morphology and locomotion, the relationship between these two traits may be as variable as the morphology itself.

### Evolution of unicapitate costovertebral morphology in Lepidosauria

While restrictive costovertebral morphology may have implications for locomotion, the evolution and function of permissive unicapitate morphology within Squamata remains unresolved. Restrictive articulations potentially function to stiffen the trunk and reduce the negative influence of locomotion on ventilation, whereas highly mobile unicapitate ribs would not mitigate locomotor forces and potentially exacerbate the conflict between these behaviors (Brainerd 2015; Claessens 2015; Brainerd et al. 2016). If some squamates seem to have re-evolved or retained more constrained costovertebral morphology, potentially for locomotion, then what benefit did permissive unicapitate ribs initially confer?

The answer may lie in the rotational freedom accessible to unicapitate joints. In this study, rotations of the first vertebral ribs of *S. merianae* during lung ventilation showed an unexpectedly high relative contribution from pump-handle, despite their hinge-like morphology (Fig. 2A, C). Relative contributions from pump-handle were also substantial in *I. iguana* and pump-handle rotations have been documented to constitute even larger relative contributions to the breathing kinematics of the savannah monitor lizard, *Varanus exanthematicus*, and the boa constrictor, *Boa constrictor*, although further investigation of both species’ costovertebral morphologies is warranted (J. G. Capano, personal observation; Cieri et al. 2018). Nevertheless, the costovertebral joints of squamates appear able to access all three potential axes of rotation, particularly pump-handle rotations, and this 3D freedom may relate to the innovative biological roles the ribs are used for in numerous squamates.

Along with lung ventilation and locomotion, ribs serve a variety of mechanical and behavioral functions throughout Squamata. Many squamates use rib rotations to affect large body shape changes for behaviors such as defensive inflation, defensive or offensive displays such as the hooding of cobras, escaping into narrow crevices, crypsis, flattening for basking, and even gliding through the air (Deban et al. 1994; Stuart-Fox et al. 2006; Young and Kardong 2010; McGuire and Dudley 2011; Socha 2011; Lillywhite 2014; Cieri 2018). Squamates are also the only extant amniote group to remain susceptible to the ancestral axial conflict between locomotion and ventilation and therefore lack substantial locomotor stamina (Brainerd and Owerkowicz 2006). Without the ability to flee for extended periods of time, the ability to fit into extremely tight spaces or deter predators through postural displays and body shape changes would have been beneficial. These motions would presumably have been less possible with restrictive bicapitate joints and evolution of permissive costovertebral joints may have enabled these new and beneficial defensive and antipredator tactics. Thus, unicapitate costovertebral morphology may have evolved to allow more rotational freedom of the ribs, particularly pump-handle rotations, to enable the large body shape changes observed throughout Squamata (Deban et al. 1994; Stuart-Fox et al. 2006; Pattishall and Cundall 2008; Young and Kardong 2010; McGuire and Dudley 2011; Socha 2011; Lillywhite 2014; Cieri 2018).

Moreover, the use of pump-handle rotations was not easily predicted *a priori* in *S. merianae*, but joint morphology was able to limit the anticipated axes of rotation used during normal ventilation. While our *a priori* predictions were accurate for bucket-handle and caliper rotations, joint morphology was not a reliable indicator of the pump-handle rotations observed. It appears that even in instances where costovertebral anatomy does provide potential axes of rotation, as in crocodilians, accurate predictions about *in vivo* pump-handle rotations remain difficult (Brocklehurst et al. 2017). Nevertheless, the findings of this study provide valuable insight for the reconstruction of the ventilation mechanics of extinct taxa. Our results suggest that analysis of single joint morphology may be a useful component of *a priori* predictions about bucket-handle and caliper rotations. Although joint morphology may not be able to predict pump-handle rotations, joint morphology analyses can serve to limit the possible axes of rotation potentially available to fossil taxa and enable more accurate reconstructions of ventilation and joint kinematics.

### Postural variation of breathing kinematics

In addition to variation in the magnitudes of the three vertebral rib rotations observed between *I. iguana* and *S. merianae*, the species breathed with substantially different postures. All members of *S. merianae* breathed with significantly higher bucket-handle angles (inflated; more positive) than *I. iguana* during both exhalation and inhalation. For average maximum inhalations, even the highest average maximum bucket-handle angle achieved by *I. iguana* barely exceeded the lowest average maximum bucket-handle angle for *S. merianae* (Fig. 7; Brainerd et al. 2016). Similarly, for average maximum exhalation, all members of *S. merianae* used higher bucket-handle angles than all but one member of *I. iguana*. Moreover, not only did *S. merianae* use more bucket-handled postures, on average they had larger magnitude changes per breath. These findings may reflect differences in thoracic architecture between these species. Argentine black and white tegus have a well-developed post-hepatic septum that partitions their viscera and lungs to increase lung volume (Klein et al. 2003b, 2003c). The presence of this post-hepatic septum even allows *S. merianae* to increase tidal volumes during low-speed locomotion, whereas neither *I. iguana* or *V. exanthematicus* showed any difference in tidal volume during exercise at similar speeds (Wang et al. 1997; Owerkowicz et al. 1999; Klein et al. 2003a). It is possible that the influence of this connective tissue sheet on the ventilation mechanics of *S. merianae* subsequently results in more adducted rib posture. Furthermore, although there are considerable differences in both vertebral rib posture and costovertebral morphology, it is difficult to assume that the restrictive joints of *S. merianae* alone would produce the increased bucket-handle postures observed. The evolutionary history of *S. merianae* may have resulted in postural variations and other mechanisms that maintain more bucket-handled rib postures and ventilation mechanics potentially beneficial for their active ecology and cursorial lifestyle.

The first sternal rib in *S. merianae* also exhibited a wide range of postures that varied between trials and individuals. Although the sternocostal synchondroses could permit rotation about all three potential axes, HA analyses revealed that the sternal ribs rotated almost entirely about a single axis within any given breath, similar to a hinge joint. Orientation of these HAs, however, displayed a wide range of variability (Fig. 8B). This variation indicates that the sternocostal joints of *S. merianae* are not actual hinge joints and suggests that the dominant axis of rotation is influenced by other factors. It is possible that, because of the intracostal joint between V1 and S1, rotations of S1 are influenced by the bucket-handle rotations of V1, similar to motions in *I. iguana* and *A. mississippiensis* (Brainerd et al. 2016; Brocklehurst et al. 2017). In this case, the wide range of HA orientations observed for S1 may be attributed to the postural variation of V1 across trials (Fig. 7). The simple sternocostal articulations of *S. merianae* may enable the sternal ribs to readily change orientation and follow vertebral rib motions about the more constrained costovertebral joints. These highly permissive sternocostal joints may also allow for extensive rotational freedom and help facilitate more 3D rib rotations and complex body shape changes associated with unicapitate costovertebral morphology.

### Concluding remarks

The diversity of breathing mechanisms observed throughout Amniota all evolved from an ancestral reliance on rib motions to ventilate the lungs. Although squamates are the only group to retain primary reliance on costal motions for ventilation, they are also the only group to not retain bicapitate ribs and have instead evolved unicapitate costovertebral morphology. The evolution of these highly permissive joints has remained a puzzling question in the evolution of Squamata. While ribs are often thought about almost exclusively in the context of ventilation, these structures also provide numerous biological functions throughout the life history of an organism. Whether helping to mitigate locomotor forces or enabling large body shape changes, the other roles ribs serve may have provided the selective context to produce the variation we see throughout Amniota and, specifically, within Squamata. It is possible that unicapitate morphology may have evolved to permit additional DOFs, particularly pump-handle rotations, to potentially enable squamates to use their ribs and trunk for defensive or behavioral purposes that would otherwise have been unavailable. Furthermore, we suggest that the ability of the sternal ribs to change their dominant axis orientation while still functioning with hinge-like motion may allow the entire rib cage the flexibility to use different axes of rotation and motions depending on the body shape and situation. The presence of more restrictive costovertebral joints in *S. merianae*, relative to the more permissive joints in *I. iguana*, may shed light onto the ecological and physiological pressures that may act upon costovertebral morphology; an increase in cursoriality and reliance on active foraging may result in more hinge-like costovertebral joints, similar to those of other active amniotes. Our findings and ability to compare rib kinematics, costovertebral morphology, and species ecology may help to infer how variation in costovertebral morphology may be related to functions outside of ventilation. Although future work is necessary to both measure rib kinematics during locomotion and to quantify maximum costovertebral ranges of motion *ex vivo*, our work also helps to test the ability of joint morphology to predict basic kinematic patterns. These insights will assist in more accurately reconstructing ventilation mechanics in fossil taxa while also providing additional context for the co-opted role of ribs in amniotes and the vexing evolution of unicapitate morphology in Squamata.
