# Erratum to: Rib Motions Don’t Completely Hinge on Joint Design: Costal Joint Anatomy and Ventilatory Kinematics in a Teiid Lizard, Salvator merianae

**DOI:** 10.1093/iob/obz010

**Published:** 2019-07-30

Rib Motions Don’t Completely Hinge on Joint Design: Costal Joint Anatomy and Ventilatory Kinematics in a Teiid Lizard, Salvator merianae

J G Capano, S Moritz, R L Cieri, L Reveret, E L Brainerd

*Integrative Organismal Biology*, Volume 1, Issue 1, 2019; oby004

In “Rib Motions Don’t Completely Hinge on Joint Design: Costal Joint Anatomy and Ventilatory Kinematics in a Teiid Lizard, Salvator merianae” (Integrative Organismal Biology, Volume 1, Issue 1, 2019, oby004, https://doi.org/10.1093/iob/oby004), in order to enable full functionality of the 3D models within the article, Figure 2 was amended to remove the panels E-H and these panels have been included instead as full 3D models 1-4 within the HTML article. Figures 3, 4, 5, 7, and 8 were inconsistent in their orientation and have been adjusted to reflect that the right ribs were marked and measured, where depicted.

